# Identification of a Transcription Factor-microRNA-Gene Coregulation Network in Meningioma through a Bioinformatic Analysis

**DOI:** 10.1155/2020/6353814

**Published:** 2020-08-07

**Authors:** Juan Wang, Yan Liang, Hui Yang, Jian-Huang Wu

**Affiliations:** ^1^Department of Infectious Diseases, Xiangya Hospital, Central South University, Changsha, China; ^2^Division of Gastroenterology and Hepatology, Mayo Clinic, Rochester Minnesota, USA; ^3^Department of Spine Surgery and Orthopaedics, Xiangya Hospital, Central South University, Changsha, China; ^4^National Clinical Research Center for Geriatric Disorders, Xiangya Hospital, Central South University, Changsha, China; ^5^Department of Radiology, The Second Xiangya Hospital, Central South University, Changsha, China

## Abstract

**Background:**

Meningioma is a prevalent type of brain tumor. However, the initiation and progression mechanisms involved in the meningioma are mostly unknown. This study aimed at exploring the potential transcription factors/micro(mi)RNAs/genes and biological pathways associated with meningioma.

**Methods:**

mRNA expressions from GSE88720, GSE43290, and GSE54934 datasets, containing data from 83 meningioma samples and eight control samples, along with miRNA expression dataset GSE88721, which had 14 meningioma samples and one control sample, were integrated analyzed. The bioinformatics approaches were used for identifying differentially expressed genes and miRNAs, as well as predicting transcription factor targets related to the differentially expressed genes. The approaches were also used for gene ontology term analysis and biological pathway enrichment analysis, construction, and analysis of protein-protein interaction network, and transcription factor-miRNA-gene coregulation network construction.

**Results:**

Fifty-six upregulated and 179 downregulated genes were identified. Thirty transcription factors able to target the differentially expressed genes were predicted and selected based on public databases. One hundred seventeen overlapping genes were identified from the differentially expressed genes and the miRNAs predicted by miRWalk. Furthermore, NF-*κ*B/IL6, PTGS2, MYC/hsa-miR-574-5p, hsa-miR-26b-5p, hsa-miR-335-5p, and hsa-miR-98-5p, which are involved in the transcription factor-miRNA-mRNA coregulation network, were found to be associated with meningioma.

**Conclusion:**

The bioinformatics analysis identified several potential molecules and relevant pathways that may represent critical mechanisms involved in the progression and development of meningioma. This work provides new insights into meningioma pathogenesis and treatments.

## 1. Introduction

Meningioma accounts for 30% of primary brain tumors, with an incidence of 5 cases per 100,000 individuals, and commonly occurs between 60 and 70 years of age, and this type of cancer originates from the cap cells of the arachnoid layer of the meninges and is usually benign [1–3]; however, malignant tumors have a high tendency to recur [3]. Symptoms are dependent on the location and can occur when the tumor presses on nearby tissues [4, 5]. Occasionally, seizures, trouble talking, dementia, vision problems, loss of bladder control, or sided weakness may occur; however, many cases never produce symptoms [6, 7]. Meningioma is more common in adults, with an incidence in females twice as much as that of men [8, 9]. Surgery is the first choice of treatment, and in recent decades, the treatment of meningioma has improved; yet, an understanding of the underlying molecular mechanisms behind its initiation and progression is lacking. Therefore, the pathogenesis of meningioma needs to be explored to improve the diagnosis, treatment, and prognosis.

Transcription factors (TFs) are DNA-binding proteins that inhibit tumors or act as oncogenes [10] and play an essential role in the regulation of gene expression, apoptosis, and cell growth [11]. Previous studies have revealed that altered expression levels of several transcription factors contribute to the aggressive development [12, 13] and malignancy [14] of meningioma.

MicroRNAs (miRNAs) are short noncoding RNAs composed of 18-25 nucleotides that regulate protein translation of mRNA [15]. Mature miRNAs recognize and bind to the 3′ untranslated region of the target mRNA, regulating target gene expression at the posttranscriptional level via mRNA translation inhibition or degradation [16]. In meningioma, numerous miRNAs act as tumor-promoting agents (oncogenes) [17] and contribute to the development of the tumor [18]. Thus, characterizing the regulatory roles of miRNAs and TFs may provide valuable information about the underlying biological processes.

Recently, bioinformatics analyses were applied to explore the underlying mechanisms of cancer to identify essential genes, noncoding RNAs, and TFs involved in initiation and progression for further experimental verification. Due to the urgent need for a better understanding of the mechanisms behind meningioma, this study aimed at exploring the potential transcription factors/microRNAs/genes and biological pathways associated with meningioma using bioinformatics approach.

## 2. Materials and Methods

### 2.1. Data Collection

We searched several keywords separated or combined, including “meningioma,” “meningiomas,” “expression profiling by array,” and “Homo sapiens” in the Gene Expression Omnibus (GEO) database (http://www.ncbi.nlm.nih.gov/geo). Four series (GSEs) were selected in this study, including the gene expression datasets GSE88720, GSE43290, and GSE54934, as well as the miRNA expression datasets GSE88721. GSE88721 contains 14 meningioma samples and one control sample, while GSE43290 contains 47 meningioma samples and four control samples. GSE54934 contains 22 meningioma samples and Table 1 three controls samples. The detailed information for each dataset is summarized in .

### 2.2. Differentially Expressed mRNA and miRNA Selection

The raw data for the gene expression datasets were collected for this analysis. The CEL files were preprocessed with Robust Multiarray Average. The combat function in the sva package was applied to remove the batch effects and unwanted sources of variation in the different datasets. Differentially expressed mRNAs and miRNAs were identified using the limma package with the empirical Bayes method. Differentially expressed genes (DEGs) were obtained from three datasets, including GSE88720, GSE43290, and GSE54934. The inclusion criteria included *P* < 0.05 and a fold change ≥1, DEMs from GSE88721 were analyzed according to the inclusion criteria *P* < 0.05 and a fold change ≥1.5.

### 2.3. Prediction of TFs and miRNA Targets and Construction of a TF-miRNA-mRNA Network

The Transcriptional Regulatory Relationships Unraveled by Sentence-based Text mining (TRRUST, http://www.grnpedia.org/trrust/) database was used to predict TFs that regulated DEGs based on the existing literature [19] and followed the methods of Chen et al. [20]. Eighty-five TFs that target DEGs were identified, and the top 30 TFs were selected for further analysis. Subsequently miRWalk 2.0 (http://zmf.umm.uni-heidelberg.de/apps/zmf/mirwalk2/miRpub.html) was used to predict the potential target genes of miRNA with validated information [21]. We identified overlapping mRNAs by intersecting the DEGs from GSE88720, GSE43290, and GSE54934 dataset with the mRNAs collected from miRWalk. Finally, a TF-miRNA-mRNA coregulation network was constructed to show the potential molecular mechanisms of meningioma initiation and progression. Cytoscape was used to visualize the interactions between the TF-miRNA-mRNA coregulation networks.

### 2.4. Function Enrichment Analysis and the Kyoto Encyclopedia of Genes and Genomes (KEGG) Pathway Analysis

The codifferential gene functional features in DEGs were analyzed with Metascape [22]. All statistically enriched terms (Gene Ontology/KEGG terms) were identified, and the accumulative hypergeometric *P* values and enrichment factors were calculated and used for filtering. The remaining significant terms were then hierarchically clustered into a tree based on the Kappa-statistical similarities among their gene memberships. Then a kappa score of 0.3 was applied as the threshold to cast the tree into term clusters. Finally, a subset of representative terms were selected from this cluster and converted into a network layout. Terms with a similarity score > 0.3 are linked by an edge. The network is visualized with Cytoscape.

### 2.5. Protein-Protein Interaction (PPI) Network Construction and Identification of Hub Genes

The online database STRING was applied to construct a PPI network [23]. The confidence score cutoffs were set as greater than 0.4. The Cytoscape software was then used to analyze the interactive relationship of the candidate proteins. The Molecular Complex Detection (MCODE), a plug-in used to score and find parameters that have been optimized to produce the best results for the network, was subsequently utilized to find clusters in the network. The modules in the PPI network were extracted using the Cluster Finding algorithm in MCODE with a node score cutoff of 0.1, a *k*-core of 2, a maximum depth of 100, and a degree cutoff of 2.

## 3. Results

### 3.1. DEGs and Differentially Expressed miRNAs (DEMs)

**Figure 1:**
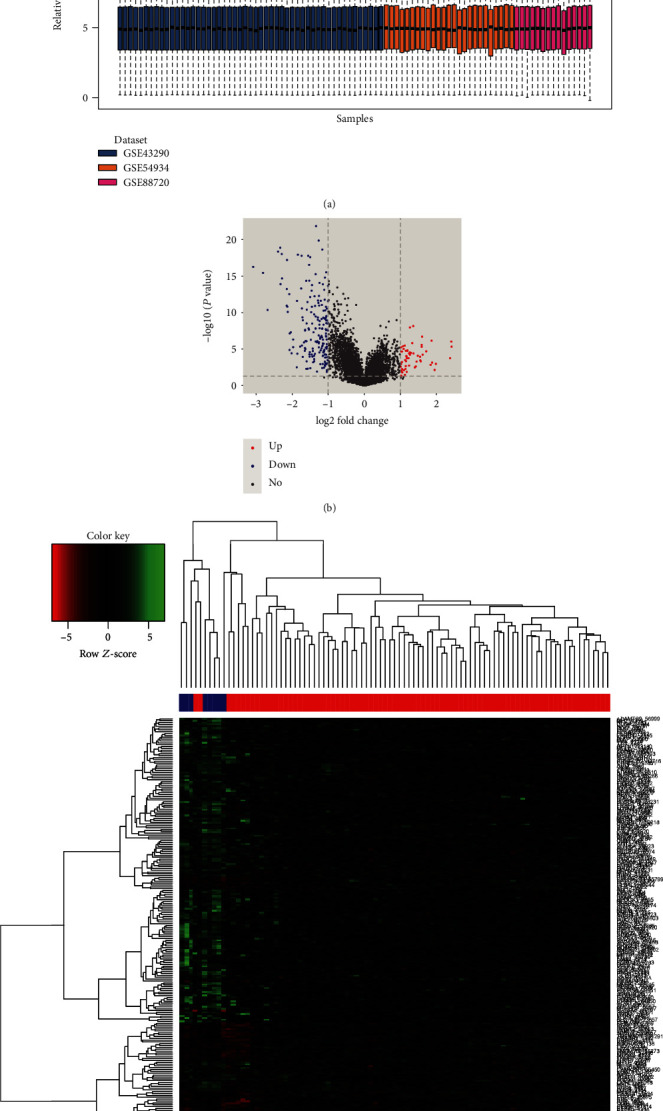
Gene expression values and cluster analysis of the meningioma samples. (a) Gene expression values of each sample after normalization. (b) Volcano plot of differentially expressed genes between meningioma and normal tissues. (c) Cluster analysis of the meningioma and normal samples based on differentially expressed genes. Lighter red in the heatmap represents high expression, while darker green indicates low expression. Black denotes medial expression.

The three mRNA expression profiles (GSE88720, GSE43290, and GSE54934), including 83 meningioma samples and eight normal tissues, were included in this study. The sva package was used in the following preprocessing to remove the batch effects (Figure 1(a)). *P* < 0.05 and ∣logFC | >1 were used as the cut-off criteria. A total of 235 DEGs (Figure 1(b), Supplementary 1) were identified, including 56 upregulated genes and 179 downregulated genes in the meningioma samples when compared to those in the normal samples. DEGs, according to the value of |logFC|, were also visualized on a heatmap (Figure 1(c)). The DEMs of GSE88721 were analyzed using the limma package. Using *P* < 0.05 and ∣logFC | ≥1.5 as the cutoff criteria, a total of 272 DEMs (Supplementary 2) were identified, including 246 upregulated and 26 downregulated miRNAs.

### 3.2. TF-miRNA-mRNA Network

**Figure 2:**
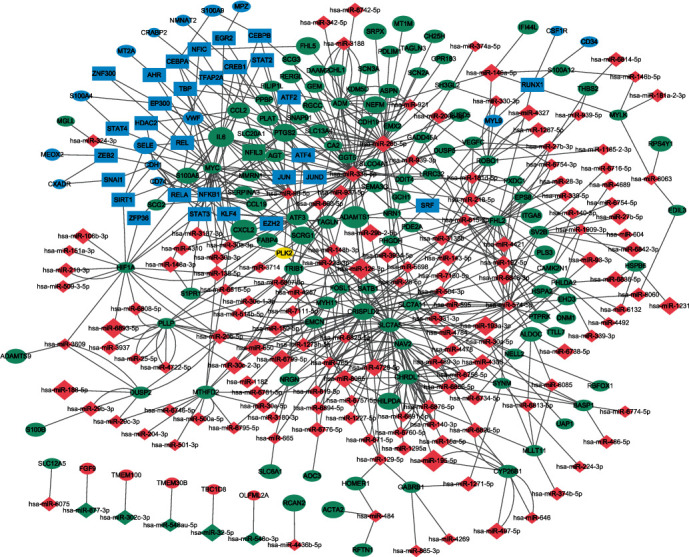
TF-miRNA-mRNA coregulation network.

**Figure 3:**
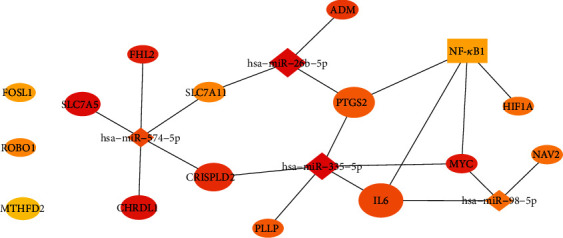
The top 20 nodes identified by CytoHubba.

Upon analysis of 235 DEGs for potential TFs in the TRRUST database, a database of transcriptional regulatory networks, 86 TFs were identified (Supplementary 3.). The top 30 enriched TFs were selected for analysis. Subsequently, miRWalk 2.0 was used to predict miRNA target genes with validated evidence. One hundred seventeen overlapping genes were identified from the DEGs in the GSE88720, GSE43290, and GSE54934 datasets and from the miRNA target genes predicted by miRWalk. A TF-miRNA-mRNA interaction network was constructed based on the TRRUST and miRWalk analyses. The TF-miRNA-mRNA network in meningioma, as visualized by Cytoscape, is shown in Figure 2. Finally, the top 20 nodes ranked by degree were identified by CytoHubba APP in Cytoscape, including 1TF (NFKB1), 4 miRNAs (hsa-miR-574-5p, hsa-miR-26b-5p, hsa-miR-335-5p, and hsa-miR-98-5p), and 15 key genes (*IL6*, *CHRDL1*, *PTGS2*, *MTHFD2*, *SLC7A11*, *ADM*, *CRISPLD2*, *ROBO1*, *FHL2*, *SLC7A5*, *MYC*, *FOSL1*, *PLLP*, *HIF1A*, and *NAV2*) (Figures 2 and 3 and Table 2).

The blue rectangle indicates TFs. The green oval indicates DEGs, and the red rhombus indicates miRNAs. The sizes of the oval and rhombus indicate the value of |logFC|.

The oval indicates DEGs, and the rhombus indicates miRNAs. The color depth and degree are consistent with the other figures.

### 3.3. Functional Enrichment of DEGs

**Figure 4:**
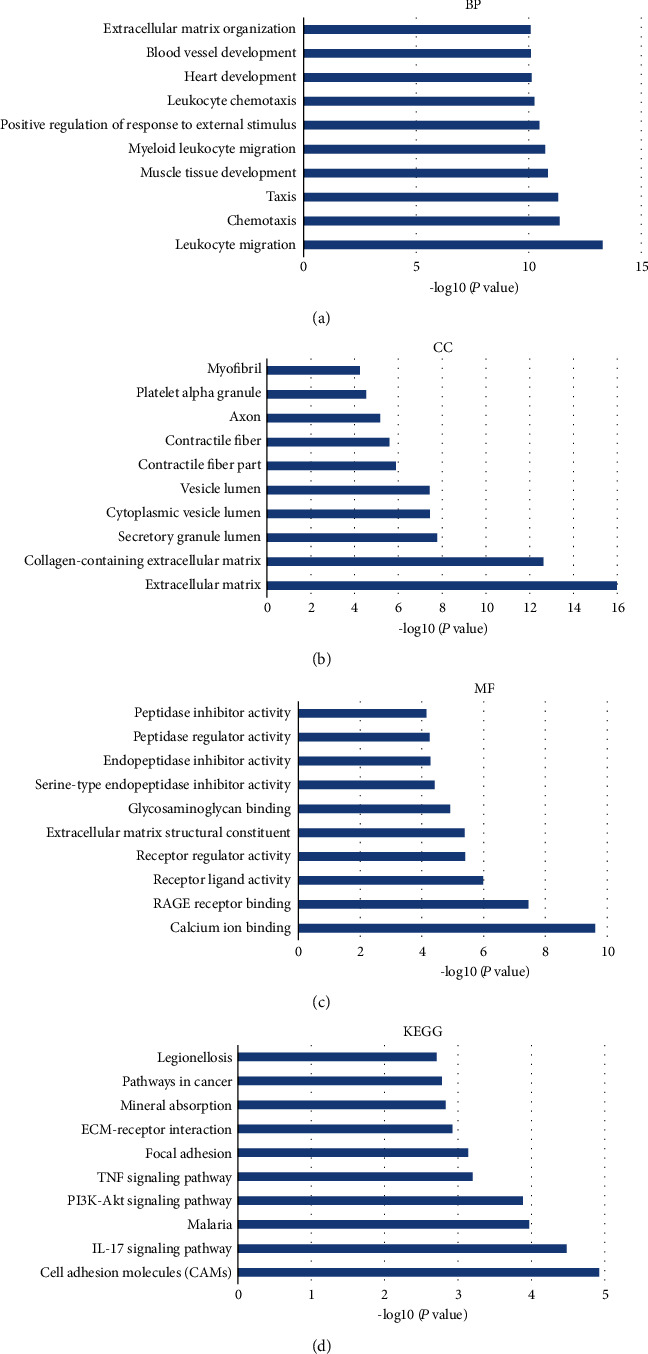
List of the GO enrichment terms for DEGs, including the top 10 clusters, and the top 10 enriched KEGG pathways.

**Figure 5:**
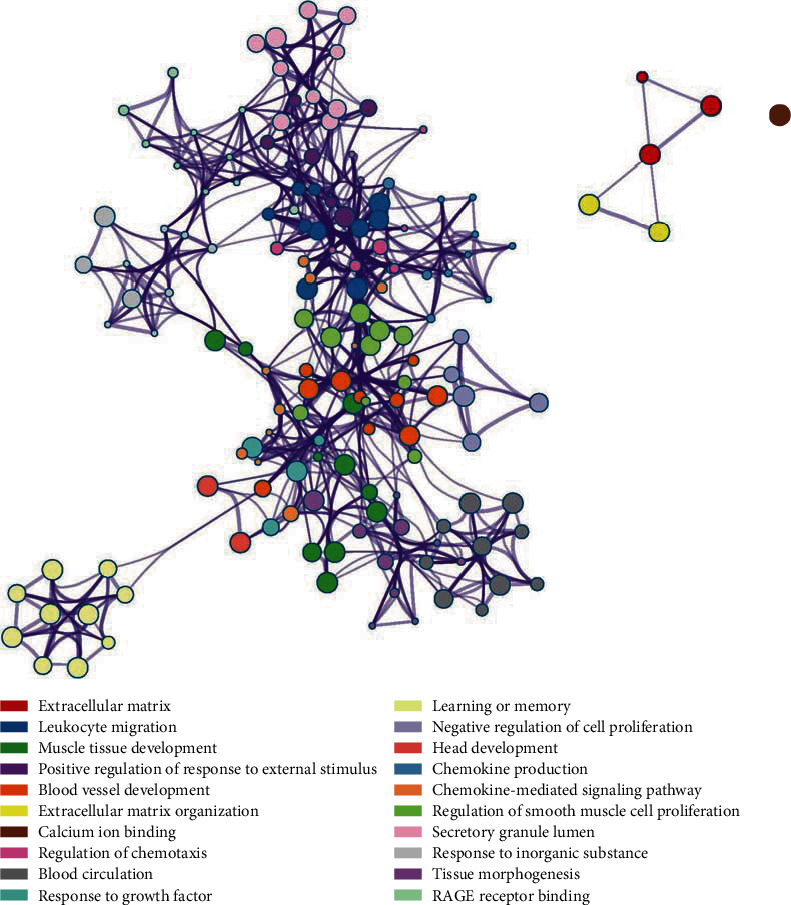
Hierarchically clustered tree of the significant terms based on Kappa-statistical similarities among their gene memberships.

GO analysis revealed 235 genes involved in several biological processes (BP), including leukocyte migration, chemotaxis, and taxis (Figure 4(a)). In terms of cellular components (CC), DEGs were mostly enriched in the extracellular matrix, collagen-containing extracellular matrix, and secretory granule lumen (Figure 4(b)). The DEGs were mainly associated with calcium ion binding, RAGE receptor binding, and receptor-ligand activity in terms of molecular functions (MF) (Figure 4(c)). Additionally, the DEGs enriched into multiple KEGG pathways, including cell adhesion molecules (CAMs), IL-17 signaling pathway, malaria, PI3K-Akt signaling pathway, and TNF signaling pathway (Figure 4(d)). A clustered tree, based on Kappa-statistical similarities of the significant terms among their gene membership, is shown in Figure 5.

### 3.4. PPI Network and Modules

**Figure 6:**
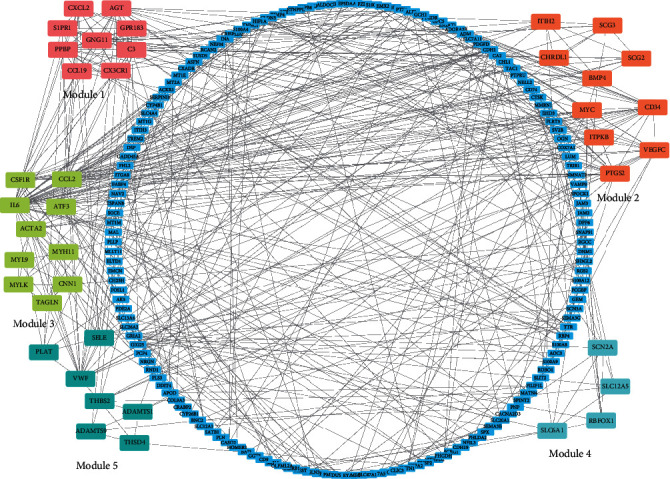
PPI network of DEGs and the top 5 modules.

**Figure 7:**
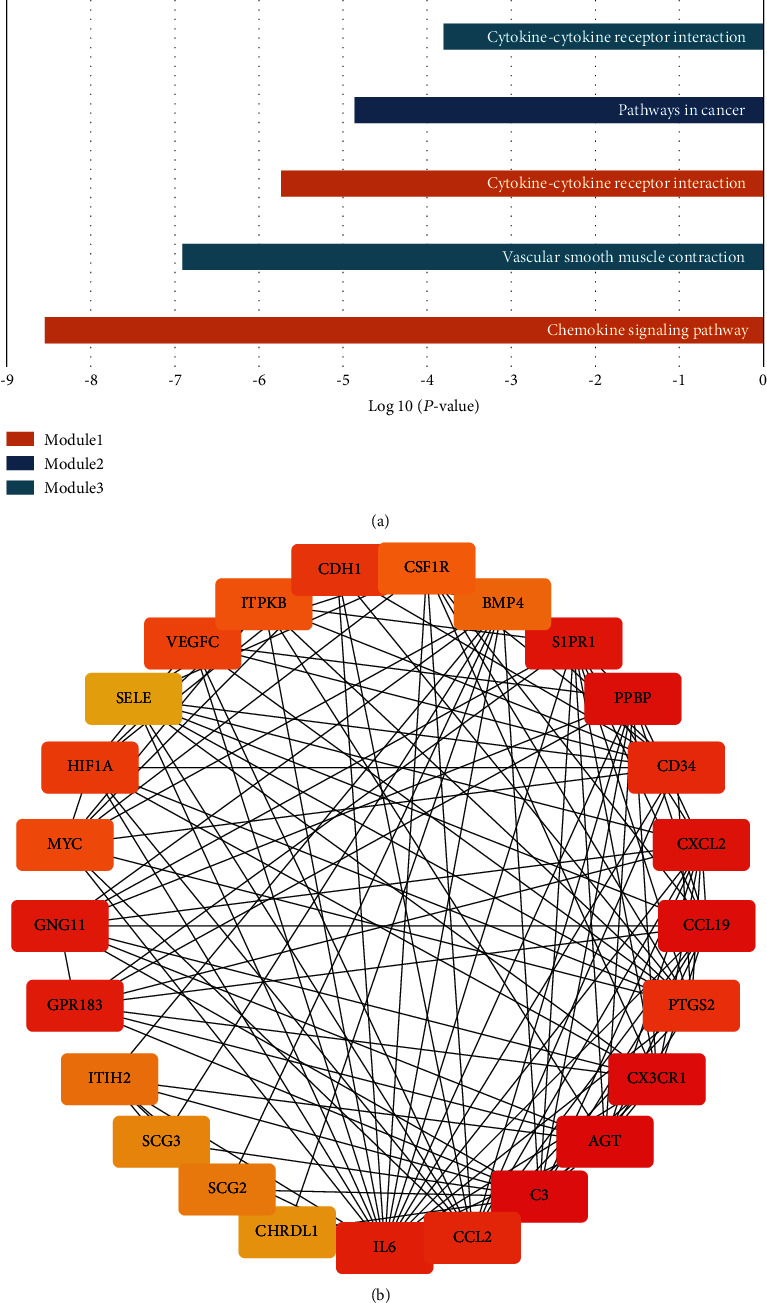
Top 5 modules genes analysis. (a) KEGG enrichment of genes within the top 5 modules. (b) The first 25 genes of the PPI network using the MCC method.

The PPIs with combined scores greater than 0.4 were selected to construct the PPI networks. The entire PPI network was analyzed using MCODE, and the top 5 modules were chosen (Figure 6). Furthermore, the KEGG pathway enrichment analysis of the module genes showed enrichment in the chemokine signaling pathway, vascular smooth muscle contraction, cytokine-cytokine receptor interaction, pathways in cancer, and cytokine-cytokine receptor interaction (Figure 7(a)). The first 25 genes were chosen by the CytoHubba plugin with the Maximal Clique Centrality (MCC) method (Figure 7(b), Table 3).

The different colors represent different functional modules of the PPI network.

## 4. Discussion

Meningioma is typically a slow-growing tumor that is derived from the meninges, the membranous layers surrounding nerve tissue, such as the brain and spinal cord [9, 17]. As this can become a debilitating disease, exploring the mechanisms of meningioma is essential to prevent recurrence and progression. Therefore, we explored the potential TFs/miRNAs/genes and biological pathways associated with meningioma using bioinformatics.

In this study, 235 DEGs were identified from three mRNA datasets, including 179 downregulated and 56 upregulated genes. TRRUST databases were used to predict 86 TFs targeting DEGs, with the top 30 enriched TFs selected for analysis. Subsequently, a TF-miRNA-target gene coregulation network was constructed to study the potential molecular mechanisms, and the top 20 nodes ranked by degree were identified, including 1 TF (NF-*κ*B), 4 miRNAs (hsa-miR-574-5p, hsa-miR-26b-5p, hsa-miR-335-5p, and hsa-miR-98-5p), and 15 key genes (IL6, CHRDL1, PTGS2, MTHFD2, SLC7A11, ADM, CRISPLD2, ROBO1, FHL2, SLC7A5, MYC, FOSL1, PLLP, HIF1A, and NAV2), which may play significant roles in meningioma. Among them, five key genes are coregulated by at least two key miRNAs or TFs, including IL6, PTGS2, SLC7A11, CRISPLD2, and MYC. Once the network was constructed, enrichment analysis and PPI analysis were performed to understand the underlying functions of these DEGs. Intriguingly, the key genes IL6, PTGS2, and MYC were also clustered into PPI module 2 or 3. Module 2 genes were enriched in pathways related to cancer, while module 3 genes were enriched in vascular smooth muscle contraction and cytokine-cytokine receptor interaction.

The results presented in this work provide an insight into the biology of meningioma. Nonetheless, most of these molecules have not been studied in meningioma. Previous studies determined that NF-*κ*B has a crucial role in inflammation and cancer initiation and progression through its ability to bind and regulate the target molecule to promote the growth of the tumor cells, suppress apoptosis, and promote angiogenesis [24, 25]. It has been reported that NF-*κ*B plays an essential role in a significant number of human cancers [26, 27], but very few studies have elucidated the function of NF-*κ*B in the pathogenesis of meningioma. NF-*κ*B was identified as key TF in this study and interacts with many DEGs and miRNAs. This may provide new insight in the role of TFs in meningioma.

The prognostic value of hsa-miR-574-5p, hsa-miR-26b-5p, hsa-miR-335-5p, and hsa-miR-98-5p in meningioma has not been reported in previous studies; however, the importance of these four miRNAs should not be underestimated. We discovered that these miRNAs are relevant to meningioma. In a previous study, it was demonstrated that knockdown of hsa-miR-574-5p expression could promote colony formation and cell invasion in colorectal cancer cells. The mechanism includes hsa-miR-574-5p negatively regulating MACC-1 expression to reach suppression in colorectal cancer [28]. However, several studies indicate that hsa-miR-574-5p is upregulated in breast cancer and pilocytic astrocytoma compared to that in control groups [29, 30]. It can be speculated that hsa-miR-574-5p has a versatile role in tumor initiation.

miR-26b plays an important role in the proliferation and metastasis of various cancer types, such as hepatocellular carcinoma and prostate cancer [31, 32], and by directly targeting SCL17 to promote apoptosis and inhibit proliferation of MCF7 cells. It was also stated in another study that upregulation of hsa-miR-26b-5p is relevant to radiation-associated breast cancer tissue samples, suggesting it may represent a radiation marker in breast cancer [33]. hsa-miR-26b-5p is known to be significantly overexpressed in both peripheral blood and tumor samples from patients with small-cell osteosarcoma, indicating that hsa-miR-26b-5p may be involved in small-cell osteosarcoma tumorigenesis [34].

Furthermore, an miRNA microarray assay in colorectal cancer tissues showed that hsa-miR-335-5p significantly associates with rectal cancer [35], and another study revealed that hsa-miR-335-5p may influence the recurrence and survival of osteosarcoma by regulating ceRNA-ceRNA interaction modules, indicating that hsa-miR-335-5p may be considered a potential novel therapeutic target in osteosarcoma [36].

Moreover, researchers found that hsa-miR-98-5p is upregulated in two NSCLC cell lines. Epigallocatechin-3-gallate (EGCG) can inhibit hsa-miR-98-5p. After the inhibition of hsa-miR-98-5p, the efficacy of cisplatin on NSCLC cell lines was enhanced. The findings indicate that hsa-miR-98-5p could be a potential target in clinical cisplatin treatment of NSCLC [37].

Our analysis also predicted MYC, PTGS2, and IL-6 in both the TF-miRNA-mRNA coregulation network and PPI network, with a high degree of interaction. This result indicates that they may act as independent factors associated with meningioma prognosis. Members of the MYC family of protooncogenes are the most commonly deregulated genes in all human cancers. MYC proteins drive an increased cellular proliferation and facilitate multiple aspects of tumor initiation and progression, thereby controlling all hallmarks of cancer [38]. A previous study has reported that MYC is a hub gene in meningioma, which is consistent with our results [39]. Another molecule predicted is PTGS2, which encodes the COX-2 enzyme and is expressed in many tumor types [40, 41]. COX-2 expression also has a strong association with tumoral grade and recurrence in meningioma [42, 43]. These findings indicate that the positive association of COX-2 with meningioma represents a potential area for therapeutic intervention with selective COX-2 inhibitors, either as an adjunct or in combination with radiation therapy. The final molecule predicted is IL-6, which belongs to the chemotactic cytokine family and correlates with occurrence, invasion, and metastasis of cancer [44]. Recent evidence suggests that, of the proinflammatory cytokines, IL-6 is a central player linking chronic inflammation to cancer by driving tumor initiation and subsequent growth and metastasis. A few studies have shown the role of IL-6 in meningioma. However, it is recently proposed that IL-6 contributes to antitumor immunity by mobilizing T cell responses as a pleiotropic cytokine, besides being a critical driver of cancer [45].

## 5. Conclusions

We constructed a TF-miRNA-mRNA coregulation network to analyze the potential molecular mechanisms of meningioma and identified 1 TF, 3 key genes, and 4 miRNAs. Though the indicated key genes are associated with meningioma, the functions of the miRNAs have not been previously evaluated. The results from the KEGG function analysis show that module genes mainly enrich in the chemokine and cytokine-signaling pathway. Among them, the NF-*κ*B/hsa-miR-98-5p/IL6 coregulation pair is predicted to be most relevant to the pathogenesis of meningioma. These TFs, miRNAs, and key genes which are related to meningioma may serve as biomarkers for the detection, prognosis, monitoring, and prediction of therapeutic responses in meningioma and may provide a novel direction for further experiments.

## Figures and Tables

**Table 1:** Characteristics of the microarray expression profile datasets.

GEO accession	Type	Platforms	Control	Meningioma	Country	Submission date
GSE43290	Expression profiling by array	GPL96	4	47	Spain	2013/1/4
GSE54934	Expression profiling by array	GPL6244	3	22	USA	2014/2/12
GSE88720	Expression profiling by array	GPL17692	1	14	Turkey	2016/10/13
GSE88721	miRNA profiling by array	GPL21572	1	14	Turkey	2016/10/13

**Table 2:** Attributes of the top 20 nodes ranked by degree.

Name	Degree	Trends	Type
NFKB1	14		TFs
hsa-miR-98-5p	17	Up	miRNA
hsa-miR-574-5p	20	Up	miRNA
hsa-miR-26b-5p	39	Up	miRNA
hsa-miR-335-5p	41	Up	miRNA
IL6	20	Down	Gene
CHRDL1	29	Down	Gene
PTGS2	18	Down	Gene
MTHFD2	13	Down	Gene
SLC7A11	15	Down	Gene
ADM	22	Down	Gene
CRISPLD2	23	Down	Gene
ROBO1	15	Down	Gene
FHL2	24	Down	Gene
SLC7A5	36	Down	Gene
MYC	27	Down	Gene
FOSL1	14	Down	Gene
PLLP	18	Down	Gene
HIF1A	17	Down	Gene
NAV2	17	Down	Gene

**Table 3:** Characteristics of the first 25 genes in the PPI network by the MCC method.

ID	degree_layout	MCODE_Cluster	MCODE_Node_Status	MCODE_Score
AGT	13	MCODE 1	Clustered	8
CDH1	10		Unclustered	6.611111111
GNG11	9	MCODE 1	Seed	8
ITPKB	10	MCODE 2	Clustered	5.5
MYC	9	MCODE 2	Clustered	5.5
HIF1A	9		Unclustered	6.611111111
CD34	11	MCODE 2	Clustered	5.727272727
SCG2	6	MCODE 2	Clustered	6
SELE	9	MCODE 5	Clustered	3.636363636
SCG3	6	MCODE 2	Clustered	6
IL6	22	MCODE 3	Clustered	4.81871345
CCL2	15	MCODE 3	Clustered	4.323529412
CHRDL1	6	MCODE 2	Clustered	6
CSF1R	10	MCODE 3	Seed	5.066666667
BMP4	10	MCODE 2	Clustered	6
PPBP	11	MCODE 1	Clustered	8
C3	15	MCODE 1	Clustered	8
CXCL2	12	MCODE 1	Clustered	8
CCL19	12	MCODE 1	Clustered	8
CX3CR1	13	MCODE 1	Clustered	8
GPR183	8	MCODE 1	Clustered	8
S1PR1	11	MCODE 1	Clustered	8
PTGS2	13	MCODE 2	Seed	6.377777778
ITIH2	7	MCODE 2	Clustered	6
VEGFC	9	MCODE 2	Clustered	5.2

## Data Availability

The datasets used and/or analyzed during the current study are available from the corresponding author on reasonable request.

## Supplementary Materials

Supplementary 1 Supplemental Table 1: A total of 235 DEGs identified from three mRNA expression profiles.

Supplementary 2 Supplemental Table 2: A total of 272 DEMs were identified from GSE88721.

Supplementary 3 Supplemental Table 3: Eighty-six potential TFs of 235 DEGs predicted from the TRRUST database.
